# The possible role of the ubiquitin proteasome system in the development of atherosclerosis in diabetes

**DOI:** 10.1186/1475-2840-6-35

**Published:** 2007-10-30

**Authors:** Raffaele Marfella, Michele D' Amico, Clara Di Filippo, Mario Siniscalchi, Ferdinando Carlo sasso, Franca Ferraraccio, Francesco Rossi, Giuseppe Paolisso

**Affiliations:** 1 Department of Geriatrics and Metabolic Diseases, Second University of Naples, Italy; 2 Department of Experimental Medicine, Second University of Naples, Italy; 3 "Centro di Eccellenza Cardiovascolare" Second University of Naples, Italy; 4 Department of Biochemistry, Section of Pathology, Second University of Naples, Italy

## Abstract

We have reviewed the impact of the ubiquitin proteasome system (UPS) on atherosclerosis progression of diabetic patients. A puzzle of many pieces of evidence suggests that UPS, in addition to its role in the removal of damaged proteins, is involved in a number of biological processes including inflammation, proliferation and apoptosis, all of which constitute important characteristics of atherosclerosis. From what can be gathered from the very few studies on the UPS in diabetic cardiovascular diseases published so far, the system seems to be functionally active to a different extent in the initiation, progression, and complication stage of atherosclerosis in the diabetic people. Further evidence for this theory, however, has to be given, for instance by specifically targeted antagonism of the UPS. Nonetheless, this hypothesis may help us understand why diverse therapeutic interventions, which have in common the ability to reduce ubiquitin-proteasome activity, can impede or delay the onset of diabetes and cardiovascular diseases (CVD).

People with type 2 diabetes are disproportionately affected by CVD, compared with those without diabetes [1]. The prevalence, incidence, and mortality from all forms of CVD (myocardial infarction, cerebro-vascular disease and congestive heart failure) are strikingly increased in persons with diabetes compared with those withoutdiabetes [2]. Furthermore, diabetic patients have not benefited by the advances in the management of obesity, dyslipidemia, and hypertension that have resulted in a decrease in mortality for coronary heart disease (CHD) patients without diabetes [3]. Nevertheless, these risk factors do not fully explain the excess risk for CHD associated with diabetes [4,5]. Thus, the determinants of progression of atherosclerosis in persons with diabetes must be elucidated. Beyond the major risk factors, several studies have demonstrated that such factors, strictly related to diabetes, as insulin-resistance, post-prandial hyperglycemia and chronic hyperglycemia play a role in the atherosclerotic process and may require intervention [6,7]. Moreover, it is important to recognize that these risk factors frequently "cluster" inindividual patients and possibly interact with each other, favouring the atherosclerosis progression toward plaque instability. Thus, a fundamental question is, "which is the common soil hypothesis that may unifying the burden of all these factors on atherosclerosis of diabetic patients? Because evidences suggest that insulin-resistance, diabetes and CHD share in common a deregulation of ubiquitin-proteasome system (UPS), the major pathway for nonlysosomal intracellular protein degradation in eucaryotic cells [8,9], in this review ubiquitin-proteasome deregulation is proposed as the common persistent pathogenic factor mediating the initial stage of the atherosclerosis as well as the progression to complicated plaque in diabetic patients.

## Ubiquitin proteasome dysfunction in atherosclerosis process

**Figure 1:**
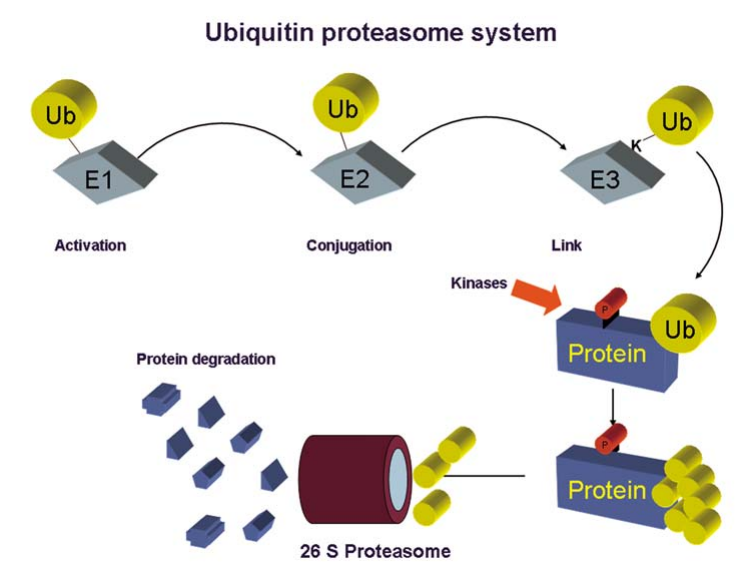
Illustration of the basic set of reactions in protein modification by ubiquitin. Processing of precursor molecules is necessary for the exposure of the conjugation site in most cases. Following its activation by the action of an E1 enzyme, the mature molecule is transferred to an E2 enzyme, which catalyzes the conjugation to the target protein. The latter action may require another "ligating" E3 enzyme. This conjugation process is balanced by deconjugation, which is mediated by a number of different enzymes. Degradation occurs in the 26S core proteasome, which contains multiple proteolytic sites within its two central rings. Peptides produced by the proteasome are released and rapidly degraded to amino acids by peptidases in the cytoplasm or transported to the endoplasmic reticulum and used in the presentation of class I antigens. The ubiquitin is not degraded but is released and reused.

**Figure 2:**
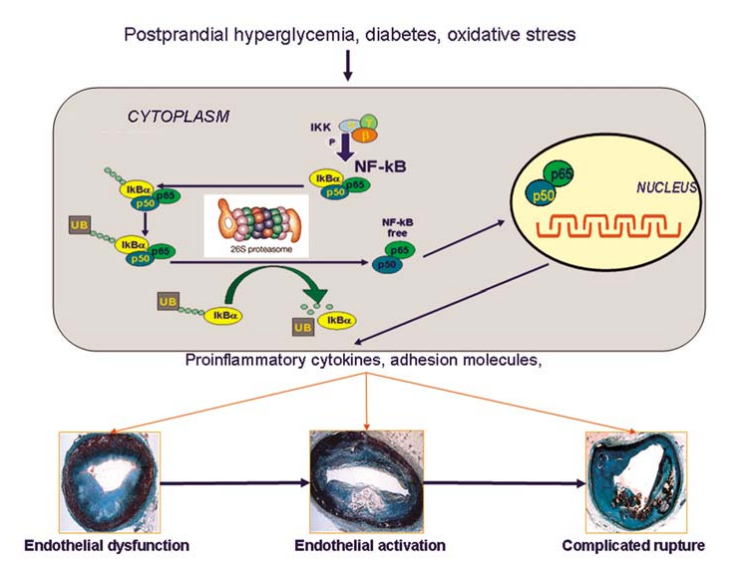
In this model, atherosclerosis process results from pathophysiological activity of UPS that may activate the NFkB inflammatory activity in the plaque macrophages. Activation pathway of NFkB, a homo- or heterodimeric transcription factor, composed by members of the Rel family of proteins. The classical example of NFkB is the heterodimer of p50 and p65, which binds to the 5'-GGGANNYYCCC-3' consensus sequence, once released from the association with an inhibitory molecule of the IkB family, primarily IkBα and IkBβ. Upon exposure of the cell to various stimuli such as increase in oxidative stress, two specific serine residues are rapidly phosphorylated by the IKK1/2 kinases. Once phosphorylated, IkBs undergo degradation via the ubiquitin-proteasome pathway in this main route of NFkB activation.

Ciechanover [10] presented in 1978 the first description of a heat-stable polypeptide that associated with an ATP-dependent proteolytic system in reticulocytes that had been previously described by Etlinger [11] in 1977. This proteolytic complex has been known by several names, including macroxyproteinase, multicatalytic proteinase complex, prosome, and, most commonly, the proteasome [12]. The UPS is responsible for the non-lysosomal degradation of the majority of intracellular proteins [13] thus playing a crucial role in the regulation of many cellular processes. The process of ubiquitination requires various enzymatic activity, involving specific proteins (i.e. E1, E2, E3) which activate and transfer polyubiquitin chains to target proteins, leading eventually to the formation of a complex which is recognized and degraded by the 26S proteasome complex [13]. This complex is composed of a 20S core particle which embodies the catalytic activity and two 19S regulatory particles. The targets of the UPS include key regulators of cell cycle and apoptosis and various transcription factors, whose intracellular levels are finely tuned in the maintenance of the optimum equilibrium for cell division, growth, differentiation, signal transduction and response to stress [14]. In addition, the UPS plays key roles in protein quality by removal of damaged, oxidized, and/or misfolded proteins [14] (Figure 1). Many of these processes are crucially involved in the onset, progression, and complication of atherosclerosis. In particular the UPS may be influenced by oxidative stress and plays a key role in the activation of nuclear factor kappa B (NFkB) [15], which has been associated with coronary [16] and carotid [17] plaque instability. Previous studies, however, indicated that the UPS could be functionally impaired under conditions of increased endogenous oxidative stress, such as diabetes and coronary artery disease [18]. Of note, it has been shown that oxidative stress can stimulate the UPS in macrophages by inducing the expression of components of its enzymatic machinery such as ubiquitin-binding proteins [19,20]. Accordingly, in cultured monocytes from patients with cerebrovascular disease has been evidenced that superoxide anion production as well as ubiquitin-proteasome activity and NFkB levels were significantly higher when compared to patients without cerebrovascular disease [21]. NFkB is normally bound to IkB in the cytosol; this binding prevents its movement into the nucleus [21]. Oxidative stress may induce ubiquitination of phosphorylated IkBs and subsequent degradation by the proteasome [22]. Degradation of IkBs results in unmasking of the nuclear localization signal of NFkB dimers, which subsequently translocates to the nucleus, where it induces the transcription of proinflammatory cytokines that play a central role in plaque instability progression [23]. Thus, increased ubiquitin-proteasome activity in plaque macrophage as consequence of oxidative stress overexpression may enhance the synthesis of NFkB in the same cell, possibly representing a crucial step in the pathophysiology of atherosclerosis progression. Foremost, a vicious circle can also develop: increased ubiquitin-proteasome activity in atherosclerotic vessels leads to increased inflammatory activity, which in turn leads to further increments of oxidative stress and consequently may increase the ubiquitinated proteins. Thus, these biological pathways including inflammation, cell proliferation, and oxidative stress, support a potential involvement of the UPS in the initiation, progression, and complication stage of atherogenesis [9] (Figure 2).

## Insulin-resistance effects on vascular biology: role of UPS activity

**Figure 3:**
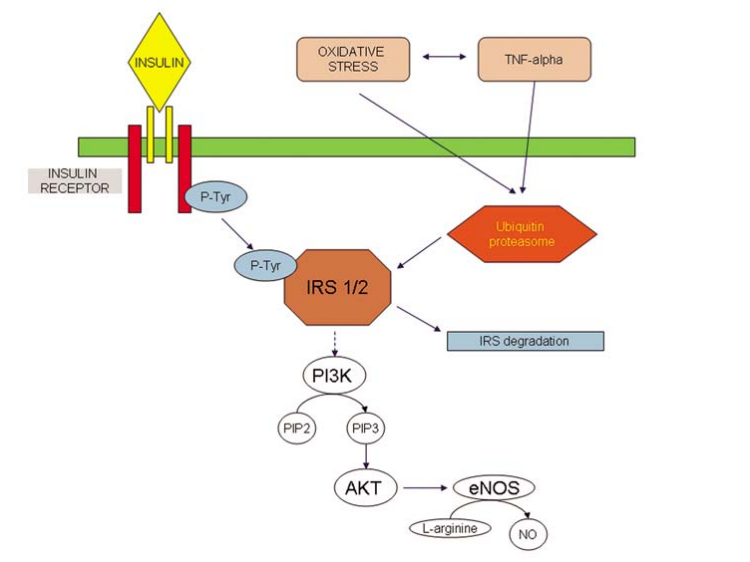
The UPS is involved in the internalization of the insulin receptor, in the control of the amount of insulin receptor substrates 1 and 2.

During the initial insulin-resistant period, or "prediabetes," the metabolic and physiological changes proceed in parallel with the commencement of silent atherosclerosis and cardiovascular complications in genetically susceptible people, before the onset of diabetes. The spectrum of atherosclerotic disturbances associated with insulin-resistance extends beyond hyperglycemia, dyslipidemia, and inflammation. Indeed, insulin receptor signalling deregulation seems to play a crucial role in causing atherosclerosis related to insulin-resistance. Binding of insulin to its receptor stimulates phosphorylation of a set of 'insulin receptor substrate' proteins, which in turn recruit and activate a lipid kinase, phosphatidylinositol 3-kinase (PI3K) [24]. Insulin, a vasodilator, increases endothelial nitric oxide (NO) production and is regulated via PI3K-dependent/Akt insulin receptor signaling [25]. In insulin-resistance, the suppression of PI3K/Akt signaling reduces NO production and impacts not only on endothelial cells but also on VSMCs, resulting in increased contraction [26]. Endothelial dysfunction is one of the earliest detectable signs in insulin-resistance, occurring even before the development of clinical manifestations. Recent reports have demonstrated that members of the ubiquitin-proteasome pathway represent new partners that have to be taken into account for the regulation of insulin action. The protein amounts of the different signaling molecules involved in insulin action are regulated by their rates of synthesis and degradation. The UPS is involved in the internalization of the insulin receptor, in the control of the amount of insulin receptor substrates 1 and 2 (IRS-1, IRS-2), and in insulin degradation [27]. Interestingly, TNF-α seems to be responsible for the insulin-resistance associated with obesity, since decreases the tyrosine kinase activity of the insulin receptor [28]. It has been demonstrated that TNF-α is able to activate the proteasome-mediated ubiquitin-dependent proteolysis. Since this proteolytic system is involved in the control of receptor-associated (tyrosine-kinase activity insulin receptor), it is postulated here that the mechanism of TNF-α-induced insulin-resistance is mediated by the activation of the UPS-dependent proteolysis [29]. Therefore, it appears that altered UPS might be one of the molecular mechanisms of insulin-resistance. In this scenario, insulin-resistance may causes suppression of PI3K/Akt signaling leading to activation of ubiquitin-proteasome proteolytic pathway [30]. Moreover, the proteasome inhibitor, PS341, could increase the level of PI3K and inhibit the downstream pathway of PI3K-dependent/Akt insulin receptor signaling, interfering with phosphorylation of Akt [31]. Thus, insulin-resistance in vascular tissue causes down-regulation of the PI3K pathway with consequential reduction of the antiatherogenic effects, interestingly this process may be influenced by UPS activity (Figure 3).

## Postprandial hyperglycemia effects on endothelial dysfunction: role of UPS activity

Repeated exposure to hyperglycemia can lead to endothelial pathology that may become irreversible over time [32]. Russell Ross [33] is highly regarded for his enunciation of the response-to-injury hypothesis, which states that endothelial damage precedes SMC migration and proliferation, deposition of intracellular and extracellular lipid, and accumulation of extracellular matrix. Subsequently, the response-to-injury hypothesis was broadened to include endothelial dysfunction and endothelial activation as the key events that precede endothelial damage and initiate the inflammatory mechanisms associated with atherosclerosis [34]. It has been demonstrated that hyperglycemic spikes induce, in both diabetic and normal subjects, an endothelial dysfunction [35,36]. Hyperglycemia may reduce both the bioavailability and the production of NO. Recent studies demonstrate that hyperglycemia induces an overproduction of superoxide by the mitochondrial electron-transport chain [37]. Superoxide overproduction is accompanied by increased NO generation, due to both endothelial NO synthase (eNOS) and inducible NO synthase (iNOS) [38]. Thesimultaneous overgeneration of NO and superoxide favors theproduction of a toxic reaction product, the peroxynitrite anion [39]. This reaction may evoke endothelial dysfunction both reducing NO availability and producing peroxynitrite anion. Moreover, the peroxynitrite anion is cytotoxic because it oxidizes sulfydryl groups in proteinsand nitrates amino acids such as tyrosine (nitrotyrosine), which results in acute endothelial dysfunction, due to the reduced NO availability [40,41]. However, recent data demonstrated that hyperglycemia reduces levels of tetrahydrobiopterin (BH4), an essential cofactor for eNOS, via 26S proteasome-mediated degradation of guanosine 5'-triphosphate cyclohydrolase I (GTPCH), which is the rate-limiting enzyme of BH4 synthesis [42]. Additionally, the authors found that hyperglycemia significantly increased superoxide anion and 3-nitrotyrosine-positive proteins and that adenoviral overexpression of superoxide dismutase (SOD) significantly attenuated hyperglycemia-induced 26S proteasome activation and GTPCH reduction. Thus, it is likely that the hyperglycemia-induced proteasome activation and GTPCH reduction are mediated by endogenous peroxynitrite anion. Moreover, administration of either the proteasome inhibitor MG132 or the SOD mimetic tempol reversed the reduction of both GTPCH and BH4 in STZ-induced diabetes mellitus in mice. Finally, MG132 abolished diabetes mellitus-induced endothelial dysfunction in vivo. Therefore, BH4 deficiency in diabetes mellitus is due to a reduction in GTPCH, an enzyme critical to BH4 synthesis, via a process that is peroxynitrite mediated and proteasome dependent. These results may have uncovered a novel mechanism underlying endothelial dysfunction in diabetic vascular diseases. On the other hand, hyperglycemic spikes may also evoked endothelial dysfunction reducing NO production through eNOS degradation. These actions of hyperglycemia may be mediated by oxidative stress, since both peroxynitrite and nitrotyrosine may induce eNOS degradation through UPS up-regulation [43]. Therefore, ubiquitin-proteasome pathways are the major proteolytic systems responsible for the regulated degradation of NOS isozymes [44]. Based in large part on the finding that eNOS degradation is blocked by lactacystin, MG132, inhibitors of UPS activity, recent study [45] evidenced that eNOS is primarily regulated by the proteasome pathway in endothelial cells. These interactions, their functional importance, and potential implication for vascular physiology and pathophysiology need to further evidences. However, the fact that eNOS is preferentially ubiquitinated suggests that the loss of endothelial function during hyperglycemia associated with a loss of eNOS activity may be due to a combination of excessive oxidative stress and UPS-dependent proteolysis [46].

## Postprandial hyperglycemia effects on endothelial activation: role of UPS activity

Repeated exposure to postprandial hyperglycemia and subsequently oxidative stress upregulation may contribute also the endothelial activation including the inflammatory and adhesive proprieties of endothelial cells through UPS activation. Previous reports evidenced a role for the UPS in the NFkB-dependent pro-inflammatory cytokine production, particularly under conditions of aggravated oxidative stress linked to hyperglycemia. In muscle wasting, NFkB activation was noted in the presence of aggravated oxidative stress and UPS overactivity [47]. Also, nitrotyrosine, which can be found in diabetic atherosclerotic lesions, has been shown to activate NFkB influencing UPS activity [48]. Various clinical studies support theevidence that an acute hyperglycemia during a hyperglycemic clamp [49] or in the postprandial state [50] can increase the production of plasma pro-inflammatory cytokines. In the continuum of the events implicated in the progression from endothelial dysfunction to endothelial activation, the hyperglycemic effects on inflammation have to be viewed together with those evidences that demonstrated an increment of the adhesion molecules under conditions of oxidative stress and UPS overactivity [51]. Adhesion molecules regulate the interaction between endothelium and leukocytes [52]. It is well known that this is considered one of the earliest and reversible stages of the process leading to atheromatous lesion. Among the various proadhesive molecules, intracellular adhesion molecule (ICAM)-1 has received particular interest. Increase in the circulating form of this molecule has been demonstrated in subjects with vascular disease [53] and with diabetes, with or without vascular disease [54]. These increases have been considered the indication of the activation of the atherogenic process. It has been demonstrated that acute hyperglycemia in both normal and diabetic subjects is a sufficient stimulus for the circulating level of ICAM-1 to increase, thus activating one of the first stages of the atherogenic process [55]. In addition to the consideration of the UPS activity as being fairly implicated on diabetic atherosclerosis, there are a number of reports on the role of 20S proteasome-proteolytic activity in regulation of adhesive properties of endothelium, for instance the activation of UPS seems to be involved in the upregulation of endothelial adhesion proteins VCAM-1 and ICAM-1 linked to oxidative stress increments [56]. Moreover, it has been shown that the protection provided by the proteasome inhibitor MLN519 is related to an anti-inflammatory effect linked with the modulation of NFkB activity, attenuation of inflammatory cytokines and cellular adhesion molecule (ICAM-1 and E-selectin) expression into the endothelial cells [57]. This scenario supports the concept that an increased UPS activity may be a mechanism linking glycemic oscillation with endothelial activation via an increased cytokine secretion as well as an increased adhesion molecule production. This pathway can lead the progression from endothelial activation, already reversible alteration, toward endothelial damage that may become irreversible alteration over time. Thus, there is some initial evidence for a pathophysiologic role of the UPS in the initial stage of atherogenesis in diabetes, which may well be related to the NFkB activation pathway.

## Influence of overt diabetes on cardiovascular events: effect of UPS deregulation on plaque destabilization

In middle-aged patients, the seven-year incidence of myocardial infarction among patients without diabetes who had preexisting CHDwas similar to that among patients with diabetes who did not have CHD, suggesting that type 2 diabetes may confer the same degree of risk as preexisting CHD [58]. The issue of the association between diabetes and CHD is likely to become more important, for two reasons. First, the incidence of type 2 diabetes is increasing among both high-risk populations and low-risk populations [59]. Second, although there has been a marked decline in the rate of death due to CHD in the overall population over the past 35 years, this has not been the case among persons with diabetes [60]. The reason for the difference is not known, but it may be that patients with diabetes have not benefited from reductions in risk factors for cardiovascular disease. This possibility is clearly not the explanation, since the reduction in the risk of CHD resulting from lipid reduction [61] and blood-pressurereduction [62] is similar for those with diabetes and those without. Moreover, the standard multitargeted intervention in the Steno-2 Studyshowed an event rate of the combined cardiovascular end point of 7% per year [63]. Although the intensified intervention involving multiple risk factors cut this event rate by half, it is still more than three times as high as in the matched background population, leaving muchroom for improvements. Thus, the diabetic status, independently from the classical cardiovascular risk factors, may influence the atherosclerotic plaque progression from stable to vulnerable, and so toward a subject susceptible to an acute coronary syndrome or sudden cardiac death based on plaque rupture, namely "cardiovascular vulnerable patients" [64]. In recent years, it has been firmly established that inflammationcontributes to plaque rupture and cardiovascular events [65]. However, little is known about the potentially unique features of this inflammatory process in diabetes. Several inflammatory markers have been identified in atherosclerotic lesions. Among them are cytokines and growth factors, which are released by activated macrophages that, together with T cells, are major cellular components in atherosclerotic lesions [66]. Cytokines increase the synthesis of platelet activating factor, stimulate lipolysis, markedly stimulate the expression of adhesion molecules, and upregulate the synthesis and cell surface expression of procoagulant activity in endothelial cells. Thus, cytokines may play a crucial role in the progression of atherosclerotic lesions toward instability. However, even if the inflammatory burden linked to diabetes not only may lead to the initiation and progression of atherosclerosis but also may contribute to plaque rupture and cardiovascular events, not much is known about inflammatory plaque differences as well as the plaque phenotype in subjects with versus those without diabetes. A thin fibrous cap and a large lipid core in association with inflammatory cell infiltration and necrotic areas, apoptosis of blood-borne and vascular cells, decrease in collagen production, and increase in collagen degradation are key characteristics of the unstable atheroma [67]. In atherectomy specimens, the cell-rich and necrotic areas are increased in de novo lesions in persons with diabetes [68]. In a series of coronary arteries examined after sudden death, the extent of the necrotic core of plaques, calcification, and healed ruptures were increased in patients with type 2 diabetes [69]. Moreover, atherosclerotic lesions from diabetic patients were characterized by higher apoptosis of VSMC, higher NFkB activation and MMP-9 levels along with a lesser interstitialcollagen content [70]. So, all this might increase the risk of future acute ischemic events precipitated by inflammatory-dependent rupture of atherosclerotic plaques. The mechanisms linking inflammation with plaque rupture in diabetes are not clear. It is well recognizedthat inflammation is one manifestation of oxidative stress [71] and the pathways that generate the mediators of inflammation, such as adhesion molecules and interleukins, are all induced by oxidative stress [72]. There are several studies demonstrating that patients with diabetes not only have increased levels of circulating markers of free radical-induced damage, but also have reduced antioxidant defenses [73]. Although these processes can be potentiated by diabetes and can contribute to the plaque rupture the molecular mechanisms linking inflammation and oxidative stress with CHD in diabetic plaques are not fully clarified. However, there is emerging evidence about the potential role of UPS also in the evolution of diabetic atherosclerotic plaques toward instability, as evidenced by the observation that the ubiquitin-proteasome pathway is required for activation of NFkB by degradation of its inhibitory IkB proteins [74]. Thus, oxidative stress the common factor underlying insulin-resistance, type 2 diabetes mellitus and CHD, may explain the presence of inflammation in all these conditions [75]. In this context, recent data suggest an interesting mechanism by which oxidative stress, increasing ubiquitin-proteasome activity, may mediate inflammatory activity in diabetic atherosclerotic plaques. Macrophages, T-lymphocytes and HDLA-DR+ inflammatory cells were more abundant in diabetic than in nondiabetic plaques and represented the major source of ubiquitin-proteasome activity, suggesting the presence of an active inflammatory reaction in diabetic lesions [48]. Moreover, in agreement with the difference in ubiquitin-proteasome staining pattern, thehistological milieu of the lesions appears different with regard to cellularity, but not in the degree of vessel stenosis, suggesting that diabetic and nondiabetic lesions are only different as regard to inflammatory burden. Of note, it has been shown that oxidative stress can stimulate the UPS in macrophages by inducing the expression of components of its enzymatic machinery such as ubiquitin-binding proteins [19]. Accordingly, in cultured monocytes from diabetic patients it has been evidenced that O2^- ^production as well as ubiquitin-proteasome activity and NFkB levels were significantly higher when compared to nondiabetic patients [48]. Thus, it has been proposed that increased ubiquitin-proteasome activity in plaque macrophage, as consequence of oxidative stress overexpression, may enhance the synthesis of NFkB in the same cell, possibly representing a crucial step in the pathophysiology of diabetic plaque instability (Figure 2). In line with this construct, the observations that the ubiquitin-proteasome activity was greater in diabetic atherosclerotic lesions as compared to nondiabetic lesions, and was associated with higher NFkB and MMP-9 levels along with a lesser interstitial collagen content, suggest that this system may have an important role in the inflammatory process ofatherosclerotic plaques of type 2 diabetic patients. However, the UPS upregulation may increases the MMP-9 expression through NFkB activation, which is known to regulate MMPs activity [76].

## Therapeutic approach

The emerging studies, on the role of UPS in the initiation and progression of atherosclerosis process, propose a number of substances which readily penetrate the cell membrane and inhibit the proteolytic function of the proteasome complex [77]. So far, the majority of data relating to the effects of proteasome inhibitors have been obtained from cancer studies [78]. Initial reports on the effects of proteasome inhibitors in cardiovascular diseases, however, indicate that proteasome inhibition might be an effective therapeutic strategy for the reduction of the proliferative phenomena of the progression stage of atherogenesis [9]. Recent data on the improvement of endothelium-dependent vasorelaxation in vitro, correlating with an increase in eNOS expression, suggest a therapeutic potential of proteasome inhibition in the early stages of atherosclerosis [45]. Finally, these substances exert a substantial anti-inflammatory effect, which was attributed to a reduction in the activity of the factor NFkB [9]. As the pathogenesis of cardiovascular events in diabetic patients involves the inflammation, the use of these drugs may be an appealing therapy. In addition to the epidemiological evidence reviewed above for the role of inflammation in diabetes-associated cardiovascular events, clinical studies of patients on cardioprotective drug regimens have revealed that many of the pharmacotherapies mediate their benefits, at least in part, through anti-inflammatory activities. This is the case most strikingly for one class of drugs that improves adipose tissue physiology and insulin sensitivity, the peroxisome proliferator-activated receptor-γ (PPARγ) agonists [79]. PPAR-γ agonist rosiglitazone, reducing the inflammation, may prevent plaque progression to an unstable phenotype in diabetic patients with asymptomatic carotid stenosis, enlisted to undergo carotid endarterectomy for extracranial high-grade (>70%) internal carotid artery stenosis [48]. The anti-inflammatory effects of glitazones are felt to be mediated partly by their beneficial effects on glycemia, but there is also evidence that glitazones may directly modulate inflammation via transcription factors such as NFKB [79]. In line with this construct, recent data show an inhibitory effect of rosiglitazone on ubiquitin-proteasome activity in diabetic lesions [48]. Indeed, at the same level of blood glucose levels, diabetic patients treated with rosiglitazone had the lowest level of ubiquitin and proteasome 20S activity, plaque inflammatory cells, cytokines, oxidative stress and MMP-9 associated with the highest content of plaque interstitial collagen. Thus, patients assigned to rosiglitazone had lesser plaque progression to an unstable phenotype compared with patients assigned to placebo. Finally, it is worth noticing that for aspirin and statins, two of the most successful drugs in cardiovascular diseases, a proteasome inhibitory effect has been described [80]. So, drugs that modulate the proteasomal degradation of proteins could become novel agents for the treatment of insulin-resistance and type 2 diabetes, as well as the pharmacological therapies targeting UPS activity may be beneficial in the treatment of vascular biology disorders associated with diabetes.

## Conclusion

We have reviewed the impact of diabetes progression on cardiovascular disease from endothelial dysfunction to plaque destabilization. A puzzle of many pieces of evidence suggests that UPS may be involved in the generation of insulin-resistance, diabetes, and cardiovascular disease. The UPS is the main route of cellular protein degradation. In addition to its role in the removal of damaged proteins, the UPS is involved in a number of biological processes including inflammation, proliferation, and apoptosis. From what can be gathered from the very few studies on the UPS in diabetic cardiovascular diseases published so far, the system seems to be functionally active to a different extent in the initiation, progression, and complication stage of atherosclerosis in the diabetic people. Further evidence for this theory, however, has to be given, for instance by specifically targeted antagonism of the UPS. Nonetheless, this hypothesis may help us understand why diverse therapeutic interventions, which have in common the ability to reduce ubiquitin-proteasome activity, can impede or delay the onset of diabetes and CVD. These early findings need further attention and confirmation, importantly with regard to therapeutic intervention, which would be indicated only if the system was clearly found to be active and pathophysiologically involved. New specific and causal inhibitors of proteasome inhibitors are available and have been successfully introduced as an adjunctive treatment option in cancer. Whether this translates to the proliferative aspects of diabetic atherosclerosis remains to be cautiously awaited.

## Competing interests

The author(s) declare that they have no competing interests.

## Authors' contributions

All authors have equally contributed in the conception and drafting of the manuscript.
